# SERCA2a tyrosine nitration coincides with impairments in maximal SERCA activity in left ventricles from tafazzin‐deficient mice

**DOI:** 10.14814/phy2.14215

**Published:** 2019-08-23

**Authors:** Jessica L. Braun, Sophie I. Hamstra, Holt N. Messner, Val A. Fajardo

**Affiliations:** ^1^ Department of Kinesiology Brock University St. Catharines ON Canada; ^2^ Centre for Bone and Muscle Health Brock University St. Catharines ON Canada

**Keywords:** Ca^2+^ regulation, phospholamban, dilated cardiomyopathy

## Abstract

The sarco/endoplasmic reticulum Ca^2+^‐ATPase (SERCA) is imperative for normal cardiac function regulating both muscle relaxation and contractility. SERCA2a is the predominant isoform in cardiac muscles and is inhibited by phospholamban (PLN). Under conditions of oxidative stress, SERCA2a may also be impaired by tyrosine nitration. Tafazzin (Taz) is a mitochondrial‐specific transacylase that regulates mature cardiolipin (CL) formation, and its absence leads to mitochondrial dysfunction and excessive production of reactive oxygen/nitrogen species (ROS/RNS). In the present study, we examined SERCA function, SERCA2a tyrosine nitration, and PLN expression/phosphorylation in left ventricles (LV) obtained from young (3‐5 months) and old (10‐12 months) wild‐type (WT) and Taz knockdown (Taz^KD^) male mice. These mice are a mouse model for Barth syndrome, which is characterized by mitochondrial dysfunction, excessive ROS/RNS production, and dilated cardiomyopathy (DCM). Here, we show that maximal SERCA activity was impaired in both young and old Taz^KD^ LV, a result that correlated with elevated SERCA2a tyrosine nitration. In addition PLN protein was decreased, and its phosphorylation was increased in Taz^KD^ LV compared with control, which suggests that PLN may not contribute to the impairments in SERCA function. These changes in expression and phosphorylation of PLN may be an adaptive response aimed to improve SERCA function in Taz^KD^ mice. Nonetheless, we demonstrate for the first time that SERCA function is impaired in LVs obtained from young and old Taz^KD^ mice likely due to elevated ROS/RNS production. Future studies should determine whether improving SERCA function can improve cardiac contractility and pathology in Taz^KD^ mice.

## Introduction

The sarco/endoplasmic reticulum Ca^2+^‐ATPase (SERCA) is a P‐type ATPase which catalyzes the active transport of Ca^2+^ ions from the cytoplasm into the sarcoplasmic reticulum (SR) (Misquitta et al., 1999; Periasamy and Huke, 2001; Palmgren and Nissen, 2011). In cardiac muscle, the predominant isoform is SERCA2a, which has been shown to be necessary in regulating muscle relaxation as well as muscle contraction by ensuring sufficient Ca^2+^ load in the SR (Misquitta et al., 1999; Periasamy and Huke, 2001; Periasamy and Kalyanasundaram, 2007). Phospholamban (PLN) is a 52 amino acid protein that, in its non‐phosphorylated state, binds to and regulates SERCA by decreasing its affinity for Ca^2+^ (Frank and Kranias, 2000; MacLennan and Kranias, 2003). When phosphorylated at serine (Ser) 16 and threonine (Thr) 17 by protein kinase A (PKA) and Ca^2+^/calmodulin‐dependent protein kinase II (CaMKII), respectively, PLN dissociates from SERCA thus restoring its affinity for Ca^2+^ ( MacLennan and Kranias, 2003).

In addition to PLN, SERCA can be regulated by reactive oxygen/nitrogen species (ROS/RNS) (Tupling et al., 1985; Viner et al., 1996; Viner et al., 1999). Structurally, the SERCA pumps are highly susceptible to oxidative and nitrosative post translational modification as they contain vulnerable cysteine, lysine, and tyrosine residues (Tupling et al., 1985; Viner et al., 1996; Viner et al., 1999). Tyrosine nitration occurs when a nitro (‐NO_2_) group is added adjacent to the hydroxyl (‐OH) group on the aromatic ring of tyrosine (Gow et al., 2004). Under conditions of oxidative stress, superoxide (O_2_^−^) and nitric oxide (NO) react to form peroxynitrite (ONOO^−^), which can then adduct to tyrosine (Pacher et al., 2007). As a result, tyrosine nitration alters protein structure and function changing the catalytic activity of many enzymes. SERCA2a specifically contains 18 tyrosine residues (Lompre et al., 1989) and has been shown to be the isoform most susceptible to tyrosine nitration leading to impairments in SERCA function (Viner et al., 1996). Others have shown that SERCA2a tyrosine nitration positively correlates with ½ relaxation time in cardiomyocytes thereby illustrating the importance of this post‐translational modification in regulating cardiac function (Lokuta et al., 2005).

Tafazzin (Taz) is a mitochondrial‐specific transacylase that regulates the formation of mature tetralinoleyl cardiolipin (L(4)CL) species (Acehan et al., 2011; Fajardo et al., 2017). Cardiolipin (CL) is an inner mitochondrial membrane phospholipid that is critical for mitochondrial function acting as an “adhesive” for the respiratory complexes (Saric et al., 2015; Zhang et al., 2016; Fajardo et al., 2017). In turn, reductions in CL and L(4)CL in Taz knockdown (Taz^KD^) mice leads to mitochondrial dysfunction and elevated ROS/RNS (He,; Phoon et al., 2012; Powers et al., 2013). Thus, it is plausible to suggest that SERCA function may be impaired in Taz^KD^ mice; however, to our knowledge, SERCA function has never been investigated. This is particularly important in their cardiac muscles since Taz^KD^ mice specifically serve as a mouse model for Barth syndrome (Acehan et al., 2011), which is a rare X‐linked recessive disease often characterized by dilated cardiomyopathy (DCM) (Acehan et al., 2011; Saric et al., 2015). Interestingly, SERCA dysfunction, SERCA2a tyrosine nitration, and PLN dysregulation have all been implicated in DCM pathology (Kelley et al., 1991; Lokuta et al., 2005; Kranias and Evangelia,; Jefferies and Towbin, 2010; Acehan et al., 2011). Therefore, in the present study, we examined SERCA function, SERCA2a tyrosine nitration, and PLN expression/phosphorylation in young (3‐5 month) and old (10‐12 month) Taz^KD^ mice.

## Methods

### Animals

Age‐matched wild‐type and Taz^KD^ male mice were obtained from a breeding colony established in Dr. Paul LeBlanc's laboratory (Brock University, St. Catharines) and the original breeding pairs were purchased from Jackson Laboratories (stock number 014648). The Taz^KD^ mice are a tetracycline inducible shRNA‐mediated Taz knockdown mouse model of Barth syndrome. Thus, to knockdown Taz and to eliminate any confounding effect of diet, both WT and Taz^KD^ mice were fed a standard chow diet supplemented with doxycycline (625 mg of dox/kg diet; Envigo TD.01306) similar to that previously described (Acehan et al., 2011). Briefly, pregnant dams and their weaned pups were fed the doxycycline diet so that the Taz^KD^ mice were Taz deficient from in utero to the time with which they were sacrificed (3‐5 months or 10‐12 months of age). All mice were allowed access to food and water *ad libitum* and were housed in an environmentally controlled room with a standard 12:12‐h light–dark cycle. All mice were euthanized via cervical dislocation while under isofluorane anesthetic, after which the left ventricles (LV) were then quickly dissected, weighed, homogenized and stored at −80°C. All animal procedures were reviewed and approved by the Brock University Animal Care and Utilization Committee (file #16‐02‐01) and carried out in accordance with the guidelines established by the Canadian Council on Animal Care.

### SERCA activity

An enzyme‐linked spectrophotometric assay was used to measure left‐ventricular SERCA activity over Ca^2+^ concentrations ranging from *p*Ca 6.9 to 4.6 in the presence of a Ca^2+ ^ionophore (A23187, Sigma C7522) as previously described (Duhamel et al., 2007).^ ^The rate of NADH disappearance, which indirectly measures ATP hydrolysis, was assessed at 340 nm and 37°C for 30 minutes using an M2 Molecular Devices MultiMode plate reader. SERCA activity was then calculated after correcting for pathlength using the extinction coefficient of NADH (6.22 mmol/L) and by subtracting ATPase activity in the presence of a SERCA specific inhibitor, cyclopiazonic acid (40 mmol/L) from total ATPase activity measured across the range of *p*Ca. All rates of SERCA activity were normalized to total protein measured with a BCA assay and the data were then fitted onto a sigmoidal dose–response curve to calculate the *p*Ca_50_ (concentration of Ca^2+^ required to elicit ½Vmax) using GraphPad Prism 8 software (GraphPad Software Inc. CA, USA). The maximal rates of SERCA activity were obtained directly from the raw data.

### Western blotting

Western blots were performed for Taz SERCA2a, PLN, and p‐PLN using TGX BioRad PreCast 4‐15% gradient gels (#4568086, BioRad). LV homogenate proteins were solubilized in 4× Laemmli buffer (#161‐0747, BioRad), separated using SDS‐PAGE, and then transferred to a polyvinylidene difluoride (PVDF) or nitrocellulose (Taz) membrane using the BioRad Transblot Turbo. All membranes were then blocked with milk (5% (w/v) in tris‐buffered saline tween [TBST]) except for membranes probing for Taz and p‐PLN, which were blocked in 3% bovine serum albumin in TBST at room temperature for 1 h before incubating overnight at 4°C with their respective primary antibodies. The Taz antibody was kindly donated from Dr. Steven Claypool (Johns Hopkins University School of Medicine) and was incubated at a 1:1000 dilution, whereas SERCA2a (MA3‐919, ThermoFisher Scientific) and PLN (MA3‐925, ThermoFisher Scientific) antibodies were incubated at a 1:2000 dilution. The p‐PLN (Ser16/Thr17; #8496, Cell Signaling Technology) antibody was incubated at a dilution of 1:5000. The membranes were washed three times in TBST after incubation before being incubated with anti‐mouse (Taz, SERCA2a and PLN) or anti‐rabbit (p‐PLN) antibodies at either a 1:5000 (Taz) or 1:10000 dilution for 1 h at room temperature. After secondary incubation the membranes were washed 3× in TBST before membranes were visualized using Clarity Western ECL Substrate (BioRad Inc) and a BioRad Chemi Doc Imager. All images were analyzed using Image Lab Software (BioRad). The blots were normalized either to a Ponceau stain (Taz), GAPDH (#2118S, Cell Signaling Technology) optical density (SERCA2a and PLN) or to total PLN (p‐PLN).

### SERCA2a tyrosine nitration

To provide an indication of SERCA oxidant damage, SERCA2a tyrosine nitration was measured using co‐immunoprecipitation. Briefly, 100 μL of SureBeads Protein G Magnetic beads (#161‐4023, BioRad) were conjugated to 3.5 μg of SERCA2a antibody (MA3‐919, ThermoFisher Scientific) in phosphate buffered saline tween (PBST). The SERCA2a‐bead complex was then incubated with 100 μg of LV homogenate protein for 1hr at room temperature. Subsequently, the beads were magnetized and supernatant discarded, then washed 3× in PBST, prior to eluting the SERCA2a protein/protein complexes with 60 μL of 1× non‐reducing Laemmli buffer at 70°C for 10 min. A western blot was then performed with the eluent using a nitrocellulose membrane and a primary antibody for nitrotyrosine (#9691, Cell Signaling Technology; 1:5000 primary, 1:10000 anti‐rabbit secondary).

### Statistical Analysis

All data are expressed as mean ± SEM. All comparisons between WT and Taz^KD^ were conducted using a two‐way ANOVA with genotype and age as main effects. A significant interaction between age and genotype was tested to determine if age would influence any effect of genotype. In the event of a significant interaction, a Sidak post hoc test enabled comparisons between genotypes within a specific age group. A Pearson's correlation was performed to examine the relationship between SERCA2a tyrosine nitration and maximal SERCA activity. Statistical significance was set to *P* < 0.05 and all data were analyzed using GraphPad Prism 8 (GraphPad Sofware Inc.).

## Results

### Body mass, tafazzin protein levels and left ventricle:body mass ratio

**Figure 1:**
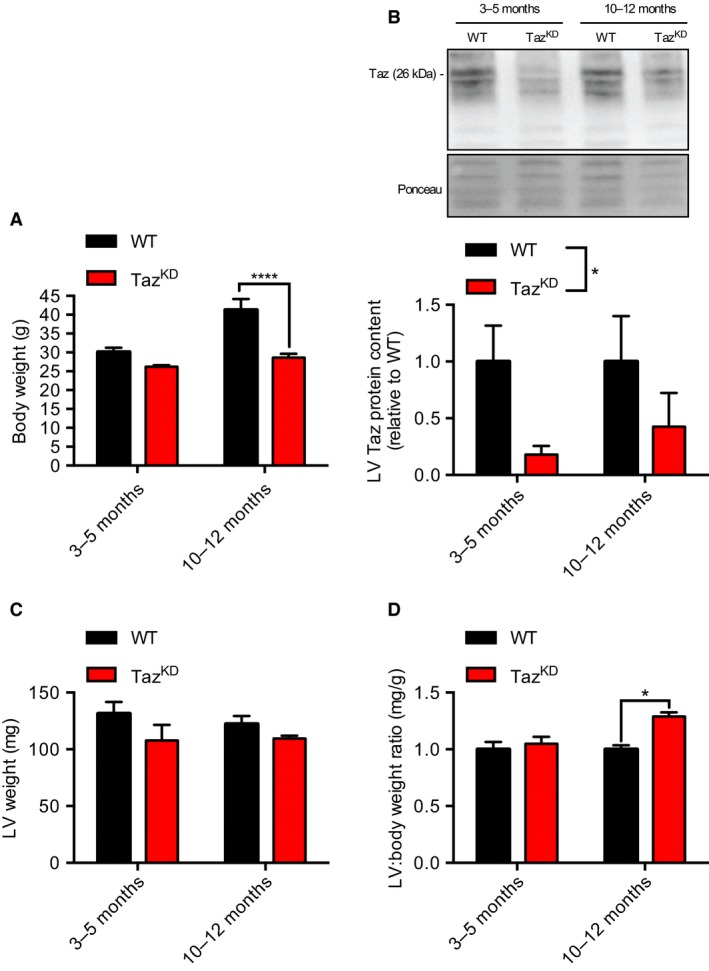
Old Taz^KD^ mice exhibit reduced growth and increased left ventricle (LV):body weight ratio. (A) Body weight (g) in young (3‐5 month) and old (10‐12 month) Taz^KD^ and WT mice. (B) LV Taz protein content expressed relative to WT, determined via Western blotting. (C) LV wet weight (mg) in young and old Taz^KD^ and WT mice. (D) LV:body weight ratio in young and old Taz^KD^ and WT mice. A two‐way ANOVA was used with age and genotype as main effects while testing for a potential interaction. For (A) and (C) a significant interaction was detected and was followed with a Sidak post hoc test that enabled comparison between genotypes within a specific age‐group. For (B) a significant main effect of genotype was detected and is depicted with an asterisk across the bar legends. *P < 0.05; ****P < 0.0001 (*n* = 5‐6 per group)

Similar to previous findings (Acehan et al., 2011), we found a significant interaction of age and genotype on body weight, whereby body weights were similar at 3‐5 months of age, but by 10‐12 months, Taz^KD^ mice were significantly smaller than WT (Fig. 1A). As expected, tafazzin levels were found to be significantly decreased (−60‐80%) in the LV obtained from Taz^KD^ mice with a significant main effect of genotype (Fig. 1B). While there were no significant differences in LV wet weight between groups (Fig. 1C), when expressed as a ratio relative to body weight we found a significant interaction indicative of a larger LV:body mass ratio but only in old Taz^KD^ mice compared with WT (Fig. 1C).

### SERCA function

**Figure 2:**
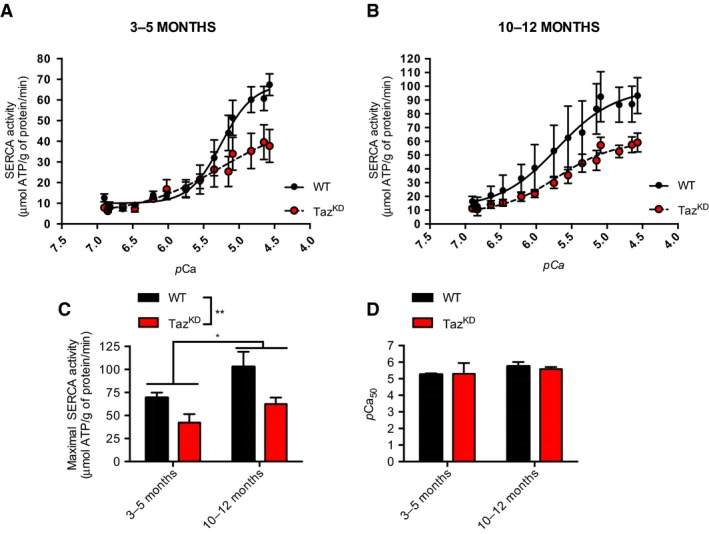
Maximal SERCA activity is reduced in LV from both young (3‐5 month) and old (10‐12 month) Taz^KD^ mice compared with WT. SERCA activity‐*p*Ca curves in (A) young and (B) old WT and Taz^KD^ mice over Ca^2+^ concentrations ranging from *p*Ca 6.9 to 4.6. (C) Maximal SERCA activity (μmol ATP/ g of protein/ min) in LV from young and old WT and Taz^KD^ mice. (D) *p*Ca_50_ is unaltered between genotypes across both age groups.  For (C), significant main effects of both age and genotype were found using a two‐way ANOVA. *P < 0.05; **P < 0.01 (*n* = 5‐6 per group)

Ca^2+^‐dependent SERCA activity was assessed to examine maximal SERCA activity and *p*Ca_50_. Our results show that both 3‐ to 5‐month old and 10‐ to 12‐month‐old Taz^KD^ mice displayed slower rates of maximal SERCA activity compared to WT with a significant main effect of genotype (Fig. 2A‐C). We also detected a significant main effect of age (Fig. 2C), suggesting that 10‐ to 12‐month‐old mice on average have faster maximal rates of SERCA activity compared with 3‐ to 5‐month‐old mice, which is likely representing the natural course of growth and development. There were no differences in the *p*Ca_50_ between groups (Fig. 2D).

### SERCA expression and tyrosine nitration

**Figure 3:**
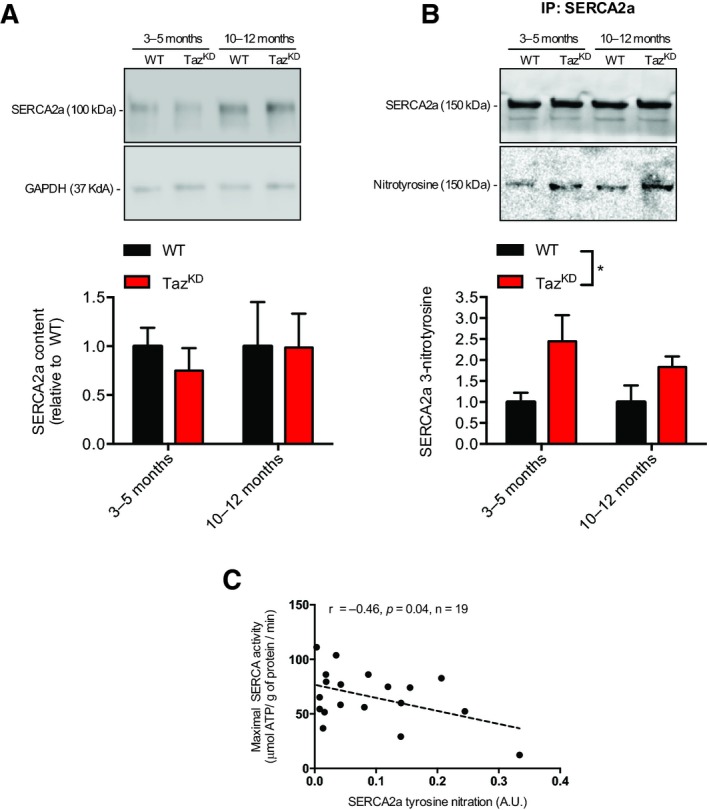
SERCA2a expression and tyrosine nitration in young (3‐5 month) and old (10‐12 month) TazKD mice compared with WT. (A) SERCA2a total protein levels determined via Western blotting was unaltered between genotypes. (B) SERCA2a tyrosine nitration was significantly elevated in LV obtained from TazKD mice, determined via Western blotting using co‐IP eluent. (C) Correlational analyses between absolute SERCA2a tyrosine nitration levels and maximal SERCA activity. For (B), a significant main effect of genotype was detected using a two‐way ANOVA (*n* = 4‐6 per group) and is depicted with an asterisk across the bar legends; and for (C), a significant negative correlation was detected using Pearson's correlational analyses, *P < 0.05

We next examined changes in the expression of SERCA2a as well as the levels of SERCA2a tyrosine nitration. SERCA tyrosine nitration occurs when peroxynitrite, formed via a reaction with nitric oxide and superoxide, attacks the tyrosine residues of the SERCA pump and can therefore be used as a marker of oxidative/nitrosative stress (Viner et al., 1996). Our results show no changes in SERCA2a expression between WT and Taz^KD^ mice across any age groups (Fig. 3A). Using co‐immunoprecpitation and non‐reducing SDS‐PAGE, we then examined the levels of SERCA2a tyrosine nitration. Under these conditions purified SERCA2a resolves at ~150 kDa (Fig. 3B). After probing for 3‐nitrotyrosine, we observed a significant increase in SERCA2a tyrosine nitration across both age groups in the Taz^KD^ mice with a significant main effect of genotype (Fig. 3B). We next examined the relationship between the absolute values of SERCA2a tyrosine nitration and maximal SERCA activity across young and old WT and Taz^KD^ mice. Our results show that SERCA2a tyrosine nitration is negatively correlated with maximal SERCA activity (Fig. 3C).

### Phospholamban expression and phosphorylation

**Figure 4:**
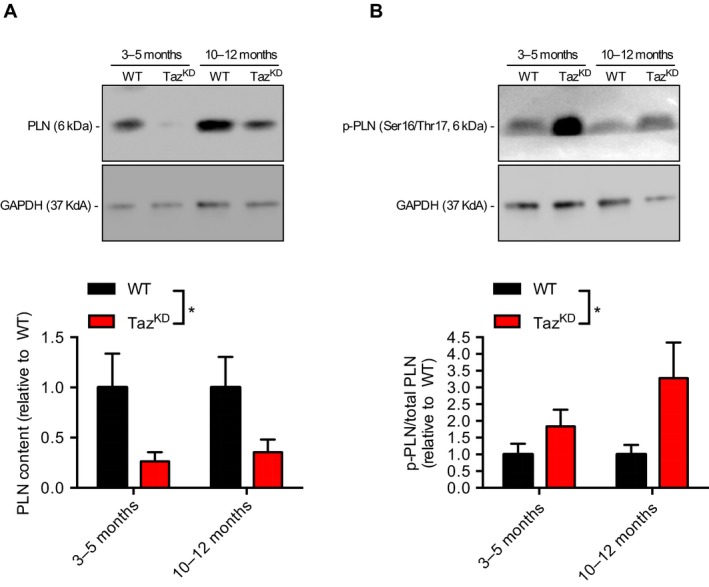
Monomeric PLN expression is decreased (A) and phosphorylated PLN is increased (B) in both young (3‐5 month) and old (10‐12 month) Taz^KD^ mice compared with WT. A two‐way ANOVA was used with age and genotype as main effects. For both (A) and (B), a significant main effect of genotype was detected and is depicted with an asterisk across the bar legends. *P < 0.05 (*n* = 4‐6 per group)

Finally, we examined the expression and phosphorylation status of PLN. Our findings show that monomeric PLN is significantly decreased in both age groups of the Taz^KD^ mice with a significant main effect of genotype (Fig. 4A). Furthermore, we observed another significant main effect of genotype indicating that PLN phosphorylation was significantly increased in Taz^KD^ LV compared to WT across both age groups (Fig. 4B).

## Discussion

In this study, we characterized LV SERCA function in young and old Taz^KD^ mice, which are distinguished by mitochondrial dysfunction and oxidative/nitrosative stress (He, 2010; Phoon et al., 2012; Powers et al., 2013). We confirmed Taz knock‐down via Western blotting in which we found a significant 60‐80% reduction in Taz protein in the LV obtained from both young and old Taz^KD^ mice. In agreement with previous results, we found that Taz^KD^ mice elicit a failure to grow, and at an older age, display an increase in LV:body weight ratios (Acehan et al., 2011). Adding to our understanding of this mouse model, our results show that maximal SERCA activity is impaired in LV from both young and old Taz^KD^ mice. These impairments in SERCA activity can be partly attributed to elevated levels of SERCA2a tyrosine nitration. In support of this, we detected a significant negative correlation between SERCA2a tyrosine nitration and maximal SERCA activity.

Our findings are consistent with previous studies that have shown that SERCA2a tyrosine nitration correlates with reductions in SERCA activity in aged rodent skeletal and cardiac muscle (Viner et al., 1999; Knyushko et al., 2005). Furthermore, in vitro experiments have previously demonstrated that incubating purified SERCA2a with increasing amounts of peroxynitrite proportionately increases tyrosine nitration ultimately impairing SERCA activity (Viner et al., 1999; Knyushko et al., 2005). While we have not conducted any experiments to determine which tyrosine residues are specifically affected in the Taz^KD^ mice, previous research has shown that Tyr294 and Tyr295 are nitrated in cardiac and skeletal muscle obtained from senescent rodents and that nitration at these residues correlates with the functional decline of SERCA (Viner et al., 1999; Knyushko et al., 2005). It is thought that nitration at Tyr294 and Tyr295 distorts helix‐helix interactions and thus hinders coordinated movements of membrane helices required for optimal rates of SERCA activity (Knyushko et al., 2005). Thus, we speculate that Tyr294 and Tyr295 are most likely nitrated in the Taz^KD^ LV, however, this should be tested more vigorously in the future.

According to the American Heart Association, DCM is one of the most common causes of heart failure (HF) (Lipshultz et al., 2019) and Ca^2+^ dysregulation has been observed in both HF and DCM with several studies reporting impairments in SERCA function (Flesch et al., 1996; Linck et al., 1996; Frank et al., 1998; Frank and Kranias, 2000). Thus, our results could also have clinical implications especially since SERCA2a tyrosine nitration has been observed in DCM hearts (Lokuta et al., 2005). As with most patients with Barth syndrome, the Taz^KD^ mice have been shown to develop DCM (Cantlay et al., 1999; Acehan et al., 2011; Phoon et al., 2012; Clarke et al., 2013) although, a recent study has reported signs of hypertrophic cardiomyopathy (HCM) (Johnson et al., 2018). Nonetheless, SERCA dysfunction has also been implicated in HCM, and thus our findings suggest that SERCA may be a viable therapeutic target for improving cardiac function and pathology in Taz^KD^ mice and potentially in those with Barth syndrome. Furthermore, Acehan et al., (2011) reported that the DCM phenotype in Taz^KD^ mice manifests only at an older age (>8 months) and cannot be observed in younger (2 month old) mice. Given that we used similar age groups, it is plausible to suggest that the impairments in SERCA function could precede the DCM phenotype in these mice and could further rationalize SERCA as a therapeutic target. While our study is limited in that we did not examine cardiac contractility or intracellular Ca^2+^, it would be interesting to determine whether improving SERCA function in Taz^KD^ mice could mitigate the cardiomyopathy.

Across several different models of DCM and HF, researchers have shown that transgenic overexpression of SERCA improves Ca^2+^ reuptake and alleviates the cardiomyopathy (He et al., 1997; Baker et al., 1998; Greene et al., 2000; Vetter et al., 2002; Lipskaia et al., 2010; Zarain‐Herzberg et al., 2014). In the context of ROS/RNS damage, protecting SERCA with chaperone proteins such as heat shock protein 70 (Hsp70) has been shown to maintain SERCA function when challenged with heat stress (Tupling et al., 2004; Fu and Tupling, 2009). Furthermore, in vivo pharmacological induction of Hsp70 with BGP15 improves skeletal muscle pathology and lifespan in a mouse model of Duchenne muscular dystrophy where ROS/RNS are important pathological mediators (Gehrig et al., 2012). While ROS/RNS damage may impair other Ca^2+^ regulatory proteins, such as ryanodine receptors and the contractile proteins actin and myosin (Bayeva and Ardehali, 2010), this is the first time SERCA dysfunction has been observed in Taz^KD^ mice and correlated with ROS/RNS damage. Thus, future studies could determine whether increasing SERCA expression or enhancing cytotoxic protection would improve SERCA function, cardiac contractility, and histopathology in Taz^KD^ mice.

While our findings indicate that SERCA2a tyrosine nitration contributes to the reductions of maximal SERCA activity in Taz^KD^ mice, our results with PLN suggest otherwise. PLN is implicated in HF and DCM with either an increase in expression or decrease in its phosphorylation (MacLennan and Kranias, 2003). In fact, several mutations to the *PLN* gene have been linked to HF and DCM (Schmitt et al., 2003; Haghighi et al., 2006; Schmitt et al., 2009; van der Zwaag et al., 2012). Here, our results show that PLN is less likely contributing to the impairments in SERCA function, given that PLN monomeric content was reduced, and its phosphorylation increased. Altogether, this would suggest that there is less PLN inhibiting the SERCA pump. Although we do not know the exact mechanisms leading to these changes in expression and phosphorylation, β‐adrenergic signaling has previously been shown to activate phosphorylation of PLN at both CaMKII and PKA sites (ser16/thr17) (Grimm and Brown, 2010). Furthermore, β‐adrenergic activation in primary cultures from murine smooth muscle cells has been shown to reduce PLN expression (McGraw et al., 2006). Importantly, elevated circulating levels of norepinephrine and epinephrine have previously been found in Taz^KD^ mice compared with WT suggestive of enhanced β‐adrenergic activation (Cole et al., 2016). Thus, the reduced expression and increased phosphorylation of PLN may be mediated by elevated β‐adrenergic stimulation, and could represent a compensatory response elicited in Taz^KD^ hearts aimed at improving SERCA function. Importantly, chronic β‐adrenergic activation can be detrimental and studies have linked it to DCM, heart failure, and early mortality (Dash et al., 2001; Grimm and Brown, 2010). Thus, it is possible that improving SERCA function in Taz^KD^ mice may lessen the need for β‐adrenergic stimulation and thus improve the physiological outcome in these mice and perhaps in Barth syndrome patients who are often prescribed beta blockers such as carvedilol (Kim et al., 2013).

In summary, we have found that maximal SERCA activity is impaired in young and old Taz^KD^ mice. This is likely due to the mitochondrial dysfunction and ensuing oxidative stress found in these mice as we observed a negative correlation between SERCA2a tyrosine nitration and maximal SERCA activity. Our findings may also have clinical implications in suggesting that SERCA may be a viable therapeutic target for the cardiomyopathy observed in Taz^KD^ mice. Thus, in the future it will be important to determine whether improving SERCA function in these mice can improve the cardiovascular outcomes.

## Conflict of interest

The authors have none to declare.
